# Late career participation of late retirees in the age of the silver tsunami: understanding the influencing mechanism of health status and employment-based health insurance participation

**DOI:** 10.1186/s12961-022-00853-y

**Published:** 2022-05-07

**Authors:** Bocong Yuan, Jiannan Li, Wenqi Liang, Junbang Lan

**Affiliations:** 1 School of Tourism Management, Sun Yat-sen University, West Xingang Rd.135, Guangzhou, 510275 China; 2 Institute of Advanced Studies in Humanities and Social Sciences, Beijing Normal University, Zhuhai, 519087 China

**Keywords:** Social health insurance, Physical functioning limitation (PFL), Instrumental activities of daily living (IADLs), Labour participation, Retirement, Delayed retirement initiative

## Abstract

**Background:**

The delayed retirement initiative and population aging have led to a growing group of late retirees. However, it remains unclear whether the existing employment-based health insurance system can effectively match the recently proposed initiative and support late retirees, especially those with pre-existing function limitations. Thus, this study aims to investigate the influencing mechanism of China’s Urban Employee Basic Medical Insurance (UEBMI), physical functioning limitation (PFL) and difficulty in instrumental activities of daily living (IADLs) on labour participation of late retirees in China.

**Methods:**

This study uses data from the China Health and Retirement Longitudinal Study (CHARLS) survey, which tracks the quality of life among older adults in China (valid sample size = 5560).

**Results:**

Empirical results show that China’s employment-based health insurance (i.e. UEBMI) and health conditions (i.e. PFL and difficulty in IADLs) are positively associated with late retirees’ withdrawal from late career participation. In addition, a higher level of difficulty in IADLs could strengthen the effect of PFL on late retirees’ withdrawal from late career participation, which could be further buffered by UEBMI beneficiary status.

**Conclusion:**

In the formulation of delayed retirement policies, it is necessary to consider the influencing mechanism of the social health insurance system and health conditions on late career participation of older workers to ensure policy effectiveness.

## Background

With the aging of the population, the sustainability of national pension insurance systems is increasingly threatened, and the reform of national retirement policies is imperative. In the past few decades, European governments have enacted pension reform acts aimed at curbing early retirement and raising the standard pension age [1]. The German government reportedly plans to raise the standard pension age from 65 in 2012 to 67 after 2024 [2]. Pension regulations in Scandinavian countries also tend to increase the legal retirement age [3]. The average retirement age in France and the Netherlands rose from 60 in 2005 and 2006 to over 62 in 2012 and 2013, respectively [4]. The gap in the average retirement age between countries is narrowing, with some targeted reforms reportedly aimed at closing the gender gap in retirement ages [4]. The employment rate of older workers in Sweden has risen sharply since the late 1990s, as has the retirement age [5]. As such, late career participation has increasingly become the mainstream for older workers.

However, current policies focus more on addressing the imbalance between the sustainability of pension systems and the shortage in the labour force by implementing delayed retirement schemes, with less attention to health conditions and maintenance of working capacity of late retirees [6]. So far, research on the determinants of labour participation among late retirees is quite insufficient. Of possible determinants, health-related conditions play a critical role [7]. Dysfunction in basic physical and instrumental activities is found to have a negative effect on working state [8]. In addition, given the high incidence of diseases among older workers, health insurance is an important factor related to their out-of-pocket expenses and the corresponding financial burden [9]. In China, the Urban Employee Basic Medical Insurance (UEBMI) scheme is a payment-free and lifetime-guaranteed health insurance plan for those who have reached the legal retirement age and met the required minimum payment period. These people do not need to stay in paid jobs to pay for medical care [10]. To fill this research gap, this study will examine the influencing mechanism of physical function limitations (PFL) and difficulty in instrumental activities of daily living (IADLs) and employment-based health insurance (China’s UEBMI) on late career participation of late retirees.

This study could advance the existing literature in several aspects. First, employment-based social health insurance is recognized as important for access to healthcare and maintenance of good health and working capacity among older adults. However, current research still lacks empirical evidence to support the relationship between employment-based social health insurance and late retirees’ labour participation. This study thus intends to fill this research gap. Second, older workers are often faced with physical impairments or difficulties. However, current policies focus mainly on incentivizing late retirement or late career participation, with little attention to how the current health insurance system and physical health of older workers interact to affect their decision to stay in the labour market. This study attempts to respond to this call. Third, although the late retirement initiative has attracted attention in recent years as the number of late retirees continues to grow, the policy nexus of the employment-based social health insurance system and the delayed retirement initiative for older workers with pre-existing health conditions has not been sufficiently discussed. This study is an early empirical attempt to investigate the mechanisms influencing labour participation among late retirees.

## Literature review and hypotheses

### Associations between UEBMI, PFL, difficulty in IADLs and late career participation of late retirees

Health insurance can act as an incentive for older workers to withdraw from the labour market [11]. High health insurance subsidies can facilitate the provision of high-quality medical services for them [12]. It is found that older workers with health insurance are 21.2% more likely to leave their jobs [13]. Compared with other social medical insurance schemes in China, the UEBMI scheme has a much more extensive list of drug reimbursement and higher levels of average reimbursement rates and reimbursement limits [14, 15]. After reaching the minimum payment period (20 years for women and 25 years for men), UEBMI beneficiaries are entitled to lifetime health security (outpatient and inpatient services) with no additional payments after retirement, whereas other health insurance schemes require annual payments without a minimum payment period [16]. The higher coverage of the UEBMI can help beneficiaries reduce the economic burden of high out-of-pocket expenditures to a greater extent [15, 17]. Accordingly, late retirees of UEBMI beneficiary status are more likely to terminate their late career participation without serious concern about a shortfall in meeting out-of-pocket expenditures.

Moreover, PFL refers to the limitations in basic physical activities in daily life (e.g. running 5 km, walking up and down stairs independently) that affect work capacity and basic social interactions of older workers [18]. Physical functioning of older adults typically declines with age, often accompanied by muscle loss and joint stiffness [19] and an increased risk of falls, dementia and other chronic conditions [20]. For example, skeletal muscle dysfunction can exacerbate the degradation of limb strength and seriously impair working capacity and quality of life among older adults [21]. Physically demanding jobs can further aggravate physical functioning issues in older workers, which in turn has a negative effect on working capacity and status [22]. Thus, PFL may leave late retirees in a worse position to continue working. To avoid excessive physical burdens of work [23], late retirees with PFL may consider terminating their late career participation.

Compared with basic activities of daily living, IADLs are task-oriented and emphasize the fulfilment of more complex activities, such as ringing up to communication, taking public transportation, doing household chores and managing finances. [24]. IADLs involve motor function, physical coordination and cognitive ability [25], which greatly affect the ability to live and work independently [26]. As physical executive function and cognitive function (e.g. working memory, abstract thinking and verbal communication) decline with age [27], the odds of difficulty in IADLs may increase among older workers [28]. Difficulty in IADLs can negatively affect older workers’ working ability (e.g. decision-making, innovation, communication and emotional management at work) [29, 30] and social participation [31]. It is thus conceivable that impaired working ability due to the difficulty in IADLs may increase the willingness of late retirees to withdraw from the labour market.

- Hypothesis 1: Employment-based health insurance (i.e. UEBMI) will positively predict withdrawal from late career participation among late retirees.
- Hypothesis 2: PFL will positively predict withdrawal from late career participation among late retirees.
- Hypothesis 3: Difficulty in IADLs will positively predict withdrawal from late career participation among late retirees.

### The two-way interaction of PFL and difficulty in IADLs on late career participation of late retirees

PFL is not necessarily accompanied by difficulty in IADLs, but could interact with difficulty in IADLs to affect late retirees’ late career participation. As noted earlier, PFL manifests as limitations in basic physical activities in daily life that can greatly impact independence in daily life for older adults [18], whereas IADLs are identified as activities that extend beyond simple self-maintenance and involve motivation to explore and interact with the environment [32]. It is found that older adults with a higher level of difficulty in IADLs have worse performance in basic activities of daily living than those with a lower level of difficulty in IADLs [33]. Difficulty in IADLs may impair the performance of basic physical activities in daily life and aggravate PFL. The overlap of the difficulty in IADLs and PFL may further drive late retirees to withdraw from late career participation. As such, the effect of PFL on withdrawal from late career participation among late retirees would be strengthened if they experienced a higher level of difficulty in IADLs as well.

- Hypothesis 4: There is a two-way interaction of PFL and difficulty in IADLs on withdrawal from late career participation among late retirees, such that the relationship between PFL and withdrawal from late career participation is stronger when difficulty in IADLs is higher (versus lower).

### The three-way interaction of UEBMI, PFL and difficulty in IADLs on late career participation of late retirees

As noted earlier, China’s UEBMI can provide relatively sound health security for older workers and effectively reduce their out-of-pocket expenditure for healthcare services as compared with other health insurance schemes [34]. Although the UEBMI is mandatory, there are also cases of uninsured individuals. The current UEBMI is not perfect [35]. Its enforcement relies heavily on the stable employment relationship. Its weak restrictions on informal employment make it difficult to fully cover informal employees [36], who account for a considerable part of the workforce in China [37]. Those who are not covered by the UEBMI may participate in other social health insurance schemes, or may not even be covered by any health insurance.

**Fig. 1:**
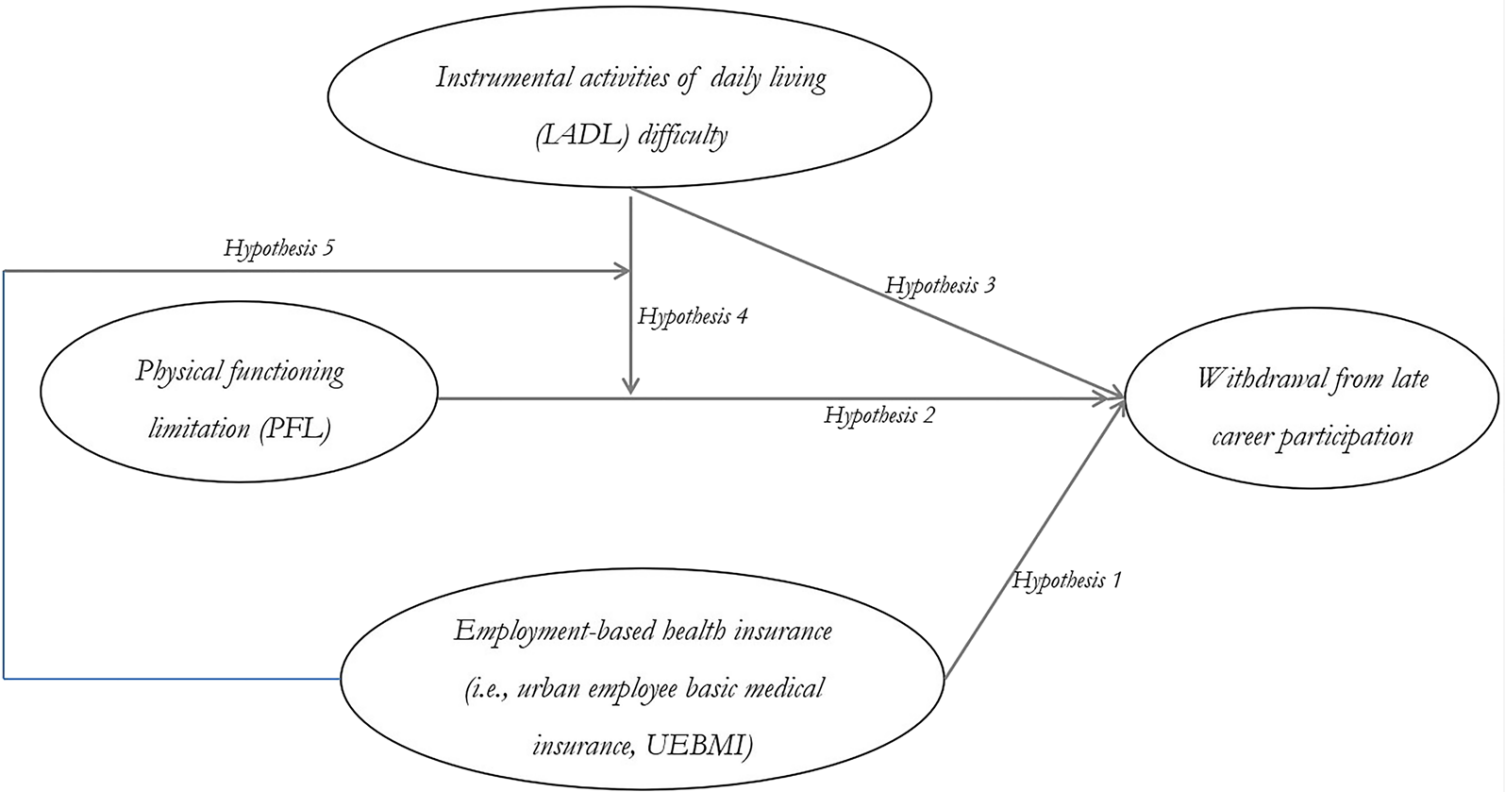
Theoretical model

Late retirees who are not covered by the UEBMI have to pay for medical treatments largely out of their own pockets and might even fall into poverty as a result [38]. However, late retirees who are beneficiaries of the UEBMI have a higher level of healthcare utilization [39], whereby those with chronic conditions can obtain cost-effective and comprehensive healthcare services [10]. Accordingly, we suppose that although difficulty in IADLs and PFL may interact to impair working capacity of late retirees, the better health security of the UEBMI can mitigate this damaging effect to some extent. The theoretical model is shown in Fig. 1.

- Hypothesis 5: There is a three-way interaction of UEBMI, PFL and difficulty in IADLs on withdrawal from late career participation among late retirees, such that the interaction of difficulty in IADLs and PFL on withdrawal from late career participation appears weaker when late retirees are insured by the UEBMI.

## Methods

### Data description

This study used data from the 2015 China Health and Retirement Longitudinal Study (CHARLS-2015) survey that investigated the health status of people aged 45 and older in China. This survey covered 150 counties (including 450 communities/villages) using stratified random sampling. Since this study focuses on the group of late retirees, only those with late retirement experience can be included in the sample (valid sample size = 5560, where all of the variables included in the regression analysis have no missing observations).

### Measures

#### Dependent variable

*Withdrawal from late career participation.* It was coded as a 0–1 binary variable. If respondents reported that they were no longer working but had ever worked after the retirement age (60 years for men and 55 years for women), they were considered as late retirees already in retirement status (i.e. having withdrawn from late career participation, coded 1). Otherwise, they were considered as late retirees who were still in working status (i.e. having not withdrawn from late career participation, coded 0).

#### Independent variables

*Employment-based social health insurance.* Respondents were asked to report whether they were currently insured by the UEBMI when surveyed. It was coded as a 0–1 binary variable (= 1 if yes; = 0 otherwise).

*PFL*. Respondents were asked to answer a seven-item question (“Have difficulty with (1) running or jogging about 1 km? (2) getting up from a chair after sitting for a long period? (3) climbing several flights of stairs without resting? (4) stooping, kneeling or crouching? (5) reaching/extending arms above shoulder level? (6) lifting/carrying weights over 5 kg? (7) picking up a small coin from a table?”). For each item, the score ranged from 1 to 4 (1 = No, I don’t have any difficulty… 4 = I cannot do it). Scores on items were averaged. The average value reflected the level of PFL. The higher average value implied a greater level of PFL*.*

*Difficulty in IADLs.* Respondents were asked to answer a six-item question (“Due to health and memory problems, do you have any difficulties with (1) doing household chores? (2) preparing hot meals? (3) shopping for groceries? (4) making phone calls? (5) taking medications? (6) managing your money, such as paying your bills, keeping track of expenses, or managing assets?”). For each item, the score ranged from 1 to 4 (1 = I have no difficulty… 4 = I cannot do it). The average value of scores on items reflected the level of difficulty in IADLs. The higher average value implied the greater level of difficulty in IADLs.

#### Covariates

*Hospital stays.* Respondents were asked whether they had experienced a hospital stay within the past year. It was coded as a 0–1 binary variable (= 1 if yes; = 0 otherwise).

*Social activity engagement.* Respondents were asked whether they had participated in at least one of the social activities listed (including “interacted with friends”, “played Ma-jong, played chess, played cards, or went to community club”, “provided help to family, friends or neighbours who do not live with you”, “went to a sport, social or other kind of club”, “took part in a community-related organization”, “did voluntary or charity work”, “cared for a sick or disabled adult who does not live with you”, “attended an educational or training course”, “stock investment”, “used the Internet”). It was coded as a 0–1 binary variable (= 1 if yes; = 0 otherwise).

*Demographics.* The effects of age, gender and marital status (coded as a 0–1 binary variable for each type of marital status listed) were controlled for. Additional details are shown in Table 1.

**Table 1:** Description of variables

Variables	Value	Frequency	Percentage (%)
Withdrawal from late career participation	1 = Yes	1323	23.77
	0 = No	4244	76.23
Physical functioning limitation	Range [1, 2]	4240	76.16
	Range [2, 3]	1124	20.19
	Range [3, 4]	203	3.65
Instrumental activities of daily living difficulty	Range [1, 2]	5017	90.12
	Range [2, 3]	407	7.29
	Range [3, 4]	143	2.59
Employment-based health insurance	1 = Yes	5272	94.70
	0 = No	295	5.30
Social activity engagement	1 = Yes	2449	43.99
	0 = No	3118	56.01
Hospital stays	1 = Yes	835	15.02
	0 = No	4726	84.98
Gender	1 = Male	2362	42.43
	2 = Female	3205	57.57
Age	1 = 55–65	3383	60.77
	2 = 66–75	1005	18.05
	3 ≥ 75	1179	21.18
Marriage status (six types)	Married	4393	78.93
	Partnered	226	4.06
	Separated	9	0.16
	Divorced	20	0.36
	Widowed	888	15.95
	Never married	30	0.54

Descriptive statistics for each variable are based on respective samples with no missing values. As a result, the sample size for each variable is different. A valid sample size in this study depends on the fact that all variables in regression analysis have no missing values. Therefore, the valid sample sizes stated in the methods section differ somewhat from values for individual variables reported in Table 1

### Analytical strategy

A stepwise regression procedure was conducted to examine relationships among variables. In the first step of regression, the main effects of the UEBMI, PFL and difficulty in IADLs on withdrawal from late career participation among late retirees were examined (hypotheses 1, 2 and 3).

$$\begin{aligned}{\text{Withdrawal}}\;{\text{from}}\;{\text{late}}\;{\text{career}}\;{\text{participation}} &= {\beta _0} + {\beta _1}\;{\text{Employment-based}}\;{\text{health}}\;{\text{insurance}} + {\beta _2}{\text{PFL}} + {\beta _3}\;{\text{Difficulty}}\;{\text{in}}\;{\text{IADLs}} \\& \quad+{\beta _4}{\text{Hospital}}\;{\text{stays}} + {\beta _5}\;{\text{Social}}\;{\text{activity}}\;{\text{engagement}} + {\beta _6}\;{\text{Age}} + {\beta _7}\;{\text{Gender}} + {\beta _8}\;{\text{Marriage}} + \varepsilon \end{aligned}$$

In the second step of regression, the two-way interaction effect of difficulty in IADLs and PFL was examined. The statistical significance of β_4_ determined whether hypothesis 4 was supported.

$$\begin{aligned} {\text{Withdrawal}}\;{\text{from}}\;{\text{late}}\;{\text{career}}\;{\text{participation}} & ={\beta _0} + {\beta _1}\;{\text{Employment-based}}\;{\text{health}}\;{\text{insurance}} + {\beta _2}\;{\text{PFL}} \\ & \quad + {\beta _3}\;{\text{Difficulty}}\;{\text{in}}\;{\text{IADLs}} + {\beta _4}\;{\text{Difficulty}}\;{\text{in}}\;{\text{IADLs}} \times {\text{PFL}} + {\beta _5}\;{\text{Hospital}}\;{\text{stays}} + {\beta _6}\;{\text{Social}}\;{\text{activity}}\;{\text{engagement}} \\ & \quad + {\beta _7}\;{\text{Age}} + {\beta _8}\;{\text{Gender}} + {\beta _9}\;{\text{Marriage}} + \varepsilon \end{aligned}$$

In the third step of regression, the three-way interaction effect of the UEBMI, PFL and difficulty in IADLs was examined. The statistical significance of β_7_ determined whether hypothesis 5 was supported.

$$\begin {aligned} {\text{Withdrawal}}\;{\text{from}}\;{\text{late}}\;{\text{career}}\;{\text{participation}} & = {\beta _0} + {\beta _1}\;{\text{Employment-based}}\;{\text{health}}\;{\text{insurance}} + {\beta _2}\;{\text{PFL}} \\ & \quad + {\beta _3}\;{\text{Difficulty}}\;{\text{in}}\;{\text{IADLs}} + {\beta _4}\;{\text{Difficulty}}\;{\text{in}}\;{\text{IADLs}} \times {\text{PFL}} + {\beta _5}\;{\text{Employment - based}}\;{\text{health}}\;{\text{insurance}} \times {\text{PFL}} \\ & \quad + {\beta _6}\;{\text{Employment-based}}\;{\text{health}}\;{\text{insurance}} \times {\text{Difficulty}}\;{\text{in}}\;{\text{IADLs}} + {\beta _7}\;{\text{Employment-based}}\;{\text{health}}\;{\text{insurance}} \\ & \quad \times {\text{Difficulty}}\;{\text{in}}\;{\text{IADLs}} \times {\text{PFL}} + {\beta _8}\;{\text{Hospital}}\;{\text{stays}} + {\beta _9}\;{\text{Social}}\;{\text{activity}}\;{\text{engagement}} + {\beta _{10}}\;{\text{Age}} + {\beta _{11}}\;{\text{Gender}} + {\beta _{12}}\;{\text{Marriage}} + \varepsilon \end{aligned}$$

## Empirical results

Table 2 shows that employment-based health insurance (0.213, *P* < 0.01, 95% CI = [0.161, 0.266]), PFL (0.107, *P* < 0.01, 95% CI = [0.084, 0.130]) and difficulty in IADLs (0.109, *P* < 0.01, 95% CI = [0.083, 0.134]) are positively associated with withdrawal from late career participation among late retirees. Thus, hypotheses 1, 2 and 3 are supported.

**Table 2:** The influence of employment-based health insurance, PFL and difficulty in IADLs on late career participation among late retirees

	Dependent variable: withdrawal from late career participation								
	Testing main effect (H1, 2, 3)			Testing two-way interaction effect (H4)			Testing three-way interaction effect (H5)		
	Coef	SE	95% CI	Coef	SE	95% CI	Coef	SE	95% CI
Main effect									
Intercept	−0.501**	0.032	[−0.565, −0.438]	−0.383 **	0.051	[−0.483, −0.284]	−0.284 **	0.054	[−0.389, −0.178]
Employment-based health insurance	0.213 **	0.027	[0.161, 0.266]	0.208 **	0.027	[0.156, 0.261]	0.105 **	0.034	[0.039, 0.171]
PFL	0.107 **	0.012	[0.084, 0.130]	0.051 *	0.022	[0.009, 0.094]	0.044 *	0.022	[0.001, 0.087]
Difficulty in IADLs	0.109 **	0.013	[0.083, 0.134]	0.020	0.034	[− 0.046, 0.086]	0.027	0.034	[−0.040, 0.093]
Two-way interaction effect									
Difficulty in IADLs × PFL				0.039 **	0.013	[0.013, 0.064]	0.040 **	0.013	[0.014, 0.066]
Employment-based health insurance × PFL							0.221 **	0.051	[0.121, 0.321]
Employment-based health insurance × Difficulty in IADLs							−0.088	0.076	[−0.237, 0.061]
Three-way interaction effect									
Employment-based health insurance × PFL × Difficulty in IADLs							−0.032 *	0.015	[−0.061, −0.003]
Covariates									
Age (years)									
55–65	Ref.			Ref.			Ref.		
66–75	0.119 **	0.010	[0.099, 0.140]	0.121 **	0.010	[0.100, 0.141]	0.120 **	0.010	[0.099, 0.140]
> 75	0.374 **	0.017	[0.340, 0.407]	0.376 **	0.017	[0.343, 0.410]	0.371 **	0.017	[0.338, 0.405]
Gender									
Male	Ref.			Ref.			Ref.		
Female	0.054**	0.011	[0.032, 0.075]	0.058 **	0.011	[0.036, 0.080]	0.061 **	0.011	[0.040, 0.083]
Marriage									
Married	Ref.			Ref.			Ref.		
Partnered	−0.009	0.023	[−0.055, 0.037]	−0.009	0.024	[−0.055, 0.037]	−0.006	0.023	[−0.052, 0.039]
Separated	0.170	0.172	[−0.167, 0.507]	0.174	0.171	[-0.161, 0.509]	0.151	0.160	[−0.163, 0.465]
Divorced	0.291**	0.110	[0.076, 0.506]	0.291 **	0.110	[0.076, 0.506]	0.292 **	0.110	[0.077, 0.507]
Widowed	0.120**	0.017	[0.088, 0.153]	0.121 **	0.017	[0.088, 0.153]	0.121 **	0.017	[0.088, 0.153]
Never married	0.040	0.076	[−0.109, 0.189]	0.045	0.075	[-0.103, 0.192]	0.050	0.075	[−0.098, 0.197]
Hospital stays past year	0.042**	0.015	[0.012, 0.072]	0.043 **	0.015	[0.013, 0.073]	0.040 **	0.015	[0.010, 0.069]
Social activity engagement	0.024*	0.010	[0.004, 0.043]	0.024 *	0.010	[0.005, 0.044]	0.023 *	0.010	[0.003, 0.042]
Number of observations	5560			5560			5555		
*F*-statistics	152.50			142.59			136.06		
*P*-values	[0.000]			[0.000]			[0.000]		

The stepwise regression analysis is based on the sample where, in each step of regression analysis, all of the variables and two- and three-way interaction terms have no missing values. *H* hypothesis, *SE* standard error. *P < 0.05, **P < 0.01

Table 2 also shows that there is a positive interactive effect between difficulty in IADLs and PFL (0.039, *P* < 0.01, 95% CI = [0.013, 0.064]) on withdrawal from the labour market among late retirees. That is, difficulty in IADLs can positively affect the relationship between PFL and withdrawal from late career participation, indicating that a higher level of difficulty in IADLs could strengthen the effect of PFL on withdrawal from late career participation among late retirees. Thus, hypothesis 4 is supported.

Moreover, in the last step of the regressions, results show that there is a negative three-way interaction of difficulty in IADLs, PFL and employment-based health insurance (−0.032, *P* < 0.01, 95% CI = [−0.061, −0.003]) on withdrawal from late career participation. That is, employment-based health insurance could negatively affect the two-way interaction between PFL and difficulty in IADLs on withdrawal from late career participation, showing that the beneficiary status of employment-based health insurance could buffer the jointly contributing effects of PFL and difficulty in IADLs on withdrawal from late career participation among late retirees. Thus, hypothesis 5 is supported.

## Discussion

### General discussion

With the aging of the population, it is imperative to explore the potential determinants of late career participation of late retirees. This study found that PFL and difficulty in IADLs may lead to termination of late career participation among late retirees. These results confirmed prior indirect evidence that physical health problems in older adults could affect their decisions about labour participation. Specifically, prior research indicated that the decline in older workers’ physical functioning, along with insomnia, inability to walk or other chronic conditions, could interfere with their daily work and life [40]. It was also shown that inability to fulfil household chores, shop or make phone calls, for example, could cause older adults to become frustrated, doubt their capability to work and live independently, and experience difficulty in maintaining good working status [30, 31]. Moreover, this study further found an overlapping effect of difficulty in IADLs and PFL on late career participation of late retirees, which has not been explored in previous studies. This overlapping effect was found to be alleviated among late retirees who were beneficiaries of the UEBMI, since this insurance scheme can significantly reduce out-of-pocket expenditures in health services, with the widest coverage and the highest level of reimbursement [15, 17].

### Practical implications

This study has some practical implications. The study empirically found that PFL and difficulty in IADLs among late retirees affected their decision about late career participation, and the overlap of these health conditions further reinforced this decision. Therefore, policy-makers need to consider characteristics and needs of specific groups of older workers in the design of delayed retirement policies to avoid the negative consequences of one-size-fits-all implementation. Policy-makers should also consider allowing older workers to voluntarily participate in retirement plans based on their health status and ability to continue working [41]. A phased retirement program may be a good solution. It provides a transition from full-time to part-time work, and eventually full retirement, by allowing older workers to work fewer hours, take on fewer responsibilities and have more flexible work schedules [42]. These measures therefore not only consider the physical condition of older workers, but also can help to meet the need for a large supply of labour.

Moreover, this study also found that the UEBMI could weaken the jointly contributing effect of PFL and difficulty in IADLs on withdrawal from late career participation among late retirees. Prior studies show that the healthcare needs of vulnerable populations who face catastrophic health expenditures are often unmet [43–46]. Therefore, the level of reimbursement needs to be improved for common diseases that cause PFL and difficulty in IADLs in the aged, such as chronic back pain, knee and hip joint replacement, cerebrovascular accident and coronary heart disease [7]. It is also necessary to incorporate geriatric rehabilitation in the UEBMI to help maintain working capacity and employment among older workers. Part of the UEBMI funds can be used to establish geriatric rehabilitation medical institutions and facilities that currently lag significantly behind those in developed countries. These practices may help older workers maintain good working conditions and relieve the pressure caused by the declining working-age population.

### Research limitations

This study still has some limitations. First, given that late retirees are still a relatively small group, the limited sample size makes it difficult to conduct a long-term follow-up in the current stage. A longer observation period is needed to capture a dynamic picture of late retirees’ participation in their future careers. Future research can provide more in-depth and broader research by the long-term tracking of older workers’ labour participation at different times and in different situations. Second, due to the lack of relevant data, it is infeasible to conduct a more fine-grained investigation on labour participation of late retirees by occupation division. Third, regarding the influence of social health insurance, PFL and difficulty in IADLs on late retirees’ withdrawal from late career participation, it remains uncertain whether such influence is temporary or permanent. Future research could clarify this issue with longer cohort studies.

## Availability of data and materials

The data are publicly available upon reasonable request and via online application to the CHARLS research team.

## Abbreviations

PFL: Physical functioning limitation
IADLs: Instrumental activities of daily living
UEBMI: Urban Employee Basic Medical Insurance
